# Correspondence Rules for *SU*(1,1) Quasidistribution Functions and Quantum Dynamics in the Hyperbolic Phase Space

**DOI:** 10.3390/e24111580

**Published:** 2022-10-31

**Authors:** Miguel Baltazar, Iván F. Valtierra, Andrei B. Klimov

**Affiliations:** Departamento de Física, Universidad de Guadalajara, Guadalajara 44420, Mexico

**Keywords:** phase space, Wigner function, *SU*(1,1) group

## Abstract

We derive the explicit differential form for the action of the generators of the SU(1,1) group on the corresponding *s*-parametrized symbols. This allows us to obtain evolution equations for the phase-space functions on the upper sheet of the two-sheet hyperboloid and analyze their semiclassical limits. Dynamics of quantum systems with SU(1,1) symmetry governed by compact and non-compact Hamiltonians are discussed in both quantum and semiclassical regimes.

## 1. Introduction

Representing non-linear quantum dynamics as an evolution of phase-space distributions not only offers an appealing visualization of sophisticated processes but also provides a convenient way to study the quantum–classical transition from the dynamical point of view [1,2,3]. The starting point for such analysis is the Liouville-like equation of motion for a quasidistribution $W_{\rho }\left(\zeta \right)$, which is a one-to-one map [4,5,6,7,8,9,10], of the density matrix $\hat{\rho }$ into a function defined on the classical phase space M,

$$\hat{\rho }\Leftrightarrow W_{\rho }\left(\zeta \right),\;\zeta \in M.$$

The structure of the phase space is determined by the symmetry group *G*—a representation that acts irreducibly in the Hilbert space H of the corresponding quantum system [11].

The evolution equation for $W_{\rho }\left(\zeta \right)$ is obtained by mapping the Schrodinger equation into the space of functions on M. To achieve this, a manageable expression for the star-product [4,5,12,13,14,15], e.g., the composition map $\hat{f}\hat{\rho }\to W_{f}\left(\zeta \right)*W_{\rho }\left(\zeta \right)$, is required if $\hat{f}$ is an arbitrary operator acting in H. Unfortunately, the general form for the star-product operation is known only for simplest groups as Heisenberg–Weyl [4,5], SU(2) [16,17,18,19,20] and some generalizations [21]. However, the maps, commonly called the correspondence rules (CR),

(1) $${\hat{c}}_{j}\hat{\rho }\to D_{L}\left({\hat{c}}_{j}\right)W_{\rho }\left(\zeta \right),\;\hat{\rho }{\hat{c}}_{j}\to D_{R}\left({\hat{c}}_{j}\right)W_{\rho }\left(\zeta \right),$$

where ${\hat{c}}_{j}$ are generators of the group *G* and $D^{L,R}\left({\hat{c}}_{j}\right)$ are some differential operators, can be obtained even for more sophisticated groups such as E(2) [22,23] and SU(3) [24]. Explicit expressions for $D_{L,R}\left({\hat{c}}_{j}\right)$ (also known as Boop [25] operators or elements of *D*-algebra [13,14,15,26,27]) are extremely useful as they allow us to obtain the phase-space evolution equations in the case when the dynamics of the system are governed by a Hamiltonian/Lindbladian that is polynomial on the group generators.

The corresponding relations are easily found for the Glauber–Sudarshan *P* and Husimi *Q* functions by using the standard coherent state machinery [26,28,29]. For arbitrary groups, these *P* and *Q* functions can be considered as representatives that are dual to each other of the *s*-parametrized quasidistributions $W_{\rho }^{\left(s\right)}\left(\zeta \right)$ with s=1 and s=−1, respectively. The situation is more involved for the self-dual Wigner function [30,31,32,33,34,35,36], $W_{\rho }^{\left(s=0\right)}\left(\zeta \right)$, which cannot be defined and treated in the same way as $W_{\rho }^{\left(\pm 1\right)}\left(\zeta \right).$ It is precisely the evolution of the Wigner function that represents the main interest due to its sensitivity to the formation of interference patterns and its specific behavior in the semiclassical limit [1,2,3,27,30,31,32,33,34,35,36].

In the present paper, we obtain the correspondence rules for quantum systems possessing SU(1,1) symmetry [37,38,39,40,41,42,43,44,45,46,47] and apply them for the analysis of phase-space dynamics generated by some non-linear (polynomial) Hamiltonians. The classical phase-space in this case is the upper sheet of the two-sheet hyperboloid. Thus, one can distinguish two types of dynamics in such a non-compact manifold: (a) a quasi-periodic evolution, generated by Hamiltonians with a discrete spectrum; and (b) a non-periodic evolution proper to continuous-spectrum systems. We analyze both types of phase-space motion in particular cases of quadratic on the group generators’ Hamiltonians. In addition, we discuss the semiclassical limit of the correspondence rules, focusing on the peculiar dynamical properties of the self-dual Wigner function.

In Section 1, we briefly overview the construction of quasidistribution functions for the SU(1,1) group. In Section 2, the correspondence rules for the Wigner function are obtained. In Section 3, we apply the correspondence rules to deduce the evolution equations for some quadratic on the group generators’ Hamiltonians; we find their exact solutions and analyze the semiclassical limit in Section 4.

## 2. The SU(1,1) Quasidistribution Functions

### 2.1. General Settings

Let us consider a quantum system with the SU(1,1) dynamic symmetry group, living in a Hilbert space H that carries an irrep labelled by the Bargman index $k=\frac{1}{2},1,\frac{3}{2},2,..$, corresponding to the positive discrete series. The group generators form the SU(1,1) algebra satisfying the following commutation relations:

(2) $$\left[{\hat{K}}_{1},{\hat{K}}_{2}\right]=-i{\hat{K}}_{0},\;\;\;\;\;\;\;\left[{\hat{K}}_{2},{\hat{K}}_{0}\right]=i{\hat{K}}_{1},\;\;\;\;\;\;\;\left[{\hat{K}}_{0},{\hat{K}}_{1}\right]=i{\hat{K}}_{2}.$$

The Hilbert space H is spanned by the eigenstates of the ${\hat{K}}_{0}$ operator,

(3) $${\hat{K}}_{0}|k,k+m\rangle =\left(k+m\right)|k,k+m\rangle \;,\;m=0,1,\ldots \;,$$

where |k,k〉 is the lowest state of the representation, defined by ${\hat{K}}_{-}|k,k\rangle =0$, ${\hat{K}}_{\pm }={\hat{K}}_{1}\pm i{\hat{K}}_{2}$. The value of the Casimir operator

(4) $$\hat{C}={\hat{K}}_{0}^{2}-{\hat{K}}_{1}^{2}-{\hat{K}}_{2}^{2},$$

is fixed to k(k−1).

Orbits of the state |k,k〉 define a set of coherent states [28]

(5) $$|n\rangle =\cosh^{-2k}\frac{\tau }{2}\sum_{m=0}^{\infty }{\left[\frac{\Gamma (m+2k)}{m!\Gamma (2k)}\right]}^{1/2}e^{-i\phi m}\tanh^{m}\frac{\tau }{2}|k,k+m\rangle \;,\;$$

labeled by the coordinates (τ,ϕ) of hyperbolic Bloch vectors in the upper sheet of the two-sheet hyperboloid

(6) $$n={\left(\cosh\tau ,\sinh\tau \cos\phi ,\sinh\tau \sin\phi \right)}^{⊤}\;,$$

The states (5) resolve the identity according to

(7) $$\begin{array}{ccc} \hat{I} & = & \frac{2k-1}{\pi }\int d^{2}\;n|n\rangle \langle n|\;, \end{array}$$

(8) $$\begin{array}{ccc} d^{2}n & = & \frac{1}{4}\sinh\tau d\tau d\phi . \end{array}$$

It is convenient to write the overlap of two coherent states in terms of the pseudo-scalar product of the respective Bloch vectors as follows:

(9) $$|\langle n|n^{\prime }\rangle {|}^{2}={\left(\frac{1+n\cdot n^{\prime }}{2}\right)}^{-2k},$$

where

(10) $$n\cdot n^{\prime }=\cosh\tau \cosh\tau^{\prime }-\cos\left(\phi -\phi^{\prime }\right)\sinh\tau \sinh\tau^{\prime }.$$

This hyperboloid can be considered as a classical phase space corresponding to our quantum system. Normalized functions f(n)≡f(τ,ϕ) on the hyperboloid can be expanded on the basis of harmonic functions,

(11) $$u_{n}^{\lambda }\left(n\right)={\left(-1\right)}^{n}\frac{\Gamma \left(\frac{1}{2}+i\lambda \right)}{\Gamma \left(\frac{1}{2}+i\lambda +n\right)}P_{-1/2+i\lambda }^{n}\left(\cosh\tau \right)e^{in\phi },$$

as follows:

(12) $$\begin{array}{ccc} f(n) & = & \sum_{n=-\infty }^{\infty }\int d\nu \left(\lambda \right)f_{\lambda n}u_{n}^{\lambda }\left(n\right),\;\;\;\;\;f_{\lambda n}=\int d^{2}nf\left(n\right)u_{n}^{*\lambda }\left(n\right), \end{array}$$

(13) $$\begin{array}{ccc} d\nu (\lambda ) & = & d\lambda \lambda \tanh(\pi \lambda ) \end{array}$$

The harmonic functions (11) are eigenfunctions of the Laplace–Beltrami operator $L^{2}$, which is a differential realization of the Casimir operator (4),

(14) $$L^{2}u_{n}^{\lambda }\left(n\right)=-\left(\lambda^{2}+\frac{1}{4}\right)u_{n}^{\lambda }\left(n\right),$$

where

(15) $$L^{2}={\tilde{k}}_{0}^{2}-{\tilde{k}}_{1}^{2}-{\tilde{k}}_{2}^{2},$$

with

(16) $${\tilde{k}}_{0}=-i\partial_{\phi },\;{\tilde{k}}_{1}=i\sin\phi \partial_{\tau }+i\cos\phi \coth\tau \partial_{\phi },\;{\tilde{k}}_{2}=-i\cos\phi \partial_{\tau }+i\sin\phi \coth\tau \partial_{\phi }$$

being differential realizations of the group generators (2). The vector field

(17) $$\tilde{k}=\left({\tilde{k}}_{0},{\tilde{k}}_{1},{\tilde{k}}_{2}\right),\;\left[\tilde{k},L^{2}\right]=0,$$

and the Bloch vector n (6) are orthogonal to each other,

(18) $$n_{0}{\tilde{k}}_{0}+n_{1}{\tilde{k}}_{1}+n_{2}{\tilde{k}}_{2}=0,$$

and satisfy the commutation relations

(19) $$\left[{\tilde{k}}_{j},n_{l}\right]=i\varepsilon_{jlm}n_{m}.$$

### 2.2. s-Parametrized Quasidistribution Functions

The identity resolution (7) allows us to define $P\left(n\right)=W^{\left(+1\right)}\left(n\right)$ and $Q\left(n\right)=W^{\left(-1\right)}\left(n\right)$ symbols of an operator $\hat{f}$ in the standard form [42,43,44,48,49,50,51,52,53,54,55],

(20) $$Q_{f}\left(n\right)=\langle n|\hat{f}|n\rangle ,$$

(21) $$\hat{f}=\frac{2k-1}{\pi }\int d^{2}nP_{f}\left(n\right)|n\rangle \langle n|,$$

so that

(22) $$\mathrm{Tr}\left(\hat{f}\hat{\varrho }\right)=\frac{2k-1}{\pi }\int d^{2}n\;P_{f}\left(n\right)Q_{\varrho }\left(n\right)\;.$$

It was observed in [45] that all elements of the *s*-parametrized family of quasidistribution functions $W^{\left(s\right)}\left(n\right)$ in the hyperbolic phase space are related to each other through a formal application of a function of the Laplace operator (15),

$$W_{f}^{\left(s\right)}\left(n\right)={\left[\Phi (L^{2})\right]}^{\frac{s^{\prime }-s}{2}}W_{f}^{\left(s^{\prime }\right)}\left(n\right),$$

where

(23) $$\Phi \left(L^{2}\right)=-\frac{\pi L^{2}}{\cos(\pi \sqrt{1/4+L^{2}})}\prod_{m=1}^{2k-2}\left[1-\frac{L^{2}}{m(m+1)}\right].$$

In particular, the self-dual Wigner symbol is obtained as a “half-way” relation between *Q* and *P* symbols,

(24) $$\begin{array}{ccc} W_{f}\left(n\right) & = & \Phi^{1/2}\left(L^{2}\right)P_{f}\left(n\right)\;=\Phi^{-1/2}\left(L^{2}\right)Q_{f}\left(n\right)\;,\\ Tr(\hat{f}\hat{\varrho }) & = & \frac{2k-1}{\pi }\int d^{2}n\;W_{f}\left(n\right)W_{\varrho }\left(n\right). \end{array}$$

In practice, the application of the $\Phi (L^{2})$ operator is carried out by using the expansions (12), e.g.,

(25) $$\begin{array}{ccc} W_{\rho }\left(n\right) & = & \frac{2}{\pi }\int d^{2}n^{\prime }\int d\nu \left(\lambda \right)\Phi^{\frac{1}{2}}\left(\lambda \right)\;P_{-\frac{1}{2}+i\lambda }\left(n^{\prime }\cdot n\right)P_{\rho }\left(n^{\prime }\right) \end{array}$$

(26) $$\begin{array}{ccc} & & \frac{2}{\pi }\int d^{2}n^{\prime }\int d\nu \left(\lambda \right)\Phi^{-\frac{1}{2}}\left(\lambda \right)\;P_{-\frac{1}{2}+i\lambda }\left(n^{\prime }\cdot n\right)Q_{\rho }\left(n^{\prime }\right)\;, \end{array}$$

where $P_{-\frac{1}{2}+i\lambda }\left(n^{\prime }\cdot n\right)$ is the conic function [56]; the function Φ(λ) is obtained from the operator (23) by substituting $L^{2}\to -\left(\lambda^{2}+\frac{1}{4}\right)$ in accordance with (14) and leading to

(27) $$\Phi \left(\lambda \right)=\frac{{\left(2k-1\right)|\Gamma \left(2k-1/2+i\lambda \right)|}^{2}}{\Gamma^{2}\left(2k\right)},$$

where Γ(z) is the Gamma function.

This also allows us to compute symbols of polynomial functions of the group generators (2). For instance, taking into account the fact that

(28) $$\begin{array}{ccc} P_{K_{j}}\left(n\right) & = & \left(k-1\right)n_{j}, \end{array}$$

(29) $$\begin{array}{ccc} P_{K_{j}^{2}}\left(n\right) & = & \frac{\left(k-1)(2k-3\right)}{2}n_{j}^{2}\pm \frac{\left(k-1\right)}{2}, \end{array}$$

where the sign “+” is for j=0 and the sign “−” is for j=1,2, one obtains

$$W_{K_{j}}\left(n\right)=\left(k-1\right)\Phi^{1/2}\left(L^{2}\right)n_{j}=\sqrt{k(k-1)}n_{j},$$

and similarly,

$$W_{K_{j}^{2}}\left(n\right)=\frac{\sqrt{k(2k+1)(k-1)(2k-3)}}{3}n_{j}^{2}\pm \frac{k(k-1)}{3}.$$

## 3. Correspondence Rules

### 3.1. Correspondence Rules for Q and P Functions

The correspondence rules (1) for *P* and *Q* functions are immediately obtained by using the basic properties of the coherent states (5). In particular, one has the following *D* algebra operators [42,43]:

(30) $$\begin{array}{ccc} {\hat{K}}_{j}\hat{\rho } & \to & W_{K_{j}\rho }^{\left(\pm 1\right)}\left(n\right)=D_{L}^{\left(\pm 1\right)}\left({\hat{K}}_{j}\right)W_{\rho }^{\left(\pm 1\right)}\left(n\right), \end{array}$$

(31) $$\begin{array}{ccc} \;\hat{\rho }{\hat{K}}_{j} & \to & W_{\rho K_{j}}^{\left(\pm 1\right)}\left(n\right)=D_{R}^{\left(\pm 1\right)}\left({\hat{K}}_{j}\right)W_{\rho }^{\left(\pm 1\right)}\left(n\right),\;j=0,1,2, \end{array}$$

which are convenient to express in vector notation as

(32) $$\begin{array}{c} D_{L,R}^{\left(s\right)}\left({\hat{K}}_{0}\right)=\left(k-\frac{s+1}{2}\right)n_{0}-s\frac{i}{2}{\left(n⋊\tilde{k}\right)}_{0}\pm \frac{1}{2}{\tilde{k}}_{0},\\ D_{L,R}^{\left(s\right)}\left({\hat{K}}_{1,2}\right)=\left(k-\frac{s+1}{2}\right)n_{1,2}-s\frac{i}{2}{\left(n⋊\tilde{k}\right)}_{1,2}\mp \frac{1}{2}{\tilde{k}}_{1,2}\;s=\pm 1, \end{array}$$

where $n_{j}$ and ${\tilde{k}}_{j}$ are the components of the pseudo-Bloch vector (6) and the vector field (16), respectively, and the deformed cross-product $n⋊\tilde{k}$ is defined as

(33) $$\begin{array}{ccc} n⋊\tilde{k} & = & \left(n_{1}{\tilde{k}}_{2}-n_{2}{\tilde{k}}_{1},n_{0}{\tilde{k}}_{2}+n_{2}{\tilde{k}}_{0},-n_{0}{\tilde{k}}_{1}-n_{1}{\tilde{k}}_{0}\right), \end{array}$$

(34) $$\begin{array}{ccc} {}[{\tilde{k}}_{j},{\left(n⋊\tilde{k}\right)}_{l}] & = & i\varepsilon_{jlm}{\left(n⋊\tilde{k}\right)}_{m}. \end{array}$$

### 3.2. Correspondence Rules for the Wigner Function

Taking into account the relation (24), we observe that

$$\begin{array}{ccc} W_{K_{j}\rho }\left(n\right) & = & \Phi^{1/2}\left(L^{2}\right)P_{K_{j}\rho }\left(n\right)=D_{L}^{\left(0\right)}\left({\hat{K}}_{j}\right)W_{\rho }\left(n\right),\\ D_{L}^{\left(0\right)}\left({\hat{K}}_{j}\right) & = & \Phi^{1/2}\left(L^{2}\right)D_{L}^{\left(+1\right)}\left({\hat{K}}_{j}\right)\Phi^{-1/2}\left(L^{2}\right). \end{array}$$

In other words, the elements of the *D* algebra for the Wigner function and *P* functions are related through a similarity transformation generated by the operator (23). This representation is quite convenient since the vector field (16) is invariant under the action of the Laplace–Beltrami operator (15). Transforming the components of the pseudo-Bloch vector (6) and making use of the orthogonality relation (18), we arrive at the following form of the CR for the Wigner function (see Appendix A):

(35) $$D_{L,R}^{\left(0\right)}\left({\hat{K}}_{j}\right)=\frac{1}{2}\left\{n_{j}A\left(L^{2}\right)-i{\left(n⋊\tilde{k}\right)}_{j}B\left(L^{2}\right)\pm {\tilde{k}}_{j}\right\},$$

where

(36) $$\begin{array}{ccc} A(L^{2}) & = & \frac{1}{2\varepsilon }\Psi \left(L^{2}\right)-\frac{\varepsilon }{2}\Psi^{-1}\left(L^{2}\right),\;B\left(L^{2}\right)=\varepsilon \Psi^{-1}\left(L^{2}\right), \end{array}$$

(37) $$\begin{array}{ccc} \Psi (L^{2}) & = & {\left[2-4\varepsilon^{2}\left(2L^{2}+1\right)+2\sqrt{1-4\varepsilon^{2}\left(2L^{2}+1\right)+16\varepsilon^{4}L^{4}}\right]}^{1/2}, \end{array}$$

and

(38) $$\varepsilon ={\left(2k-1\right)}^{-1}.$$

## 4. Evolution Equations for the Wigner Function

Applying the CR (35) to linear Hamiltonians, commonly appearing in the description of non-degenerated parametric processes, with a realization in terms of boson operators, ${\hat{K}}_{0}=\left({\hat{a}}^{†}\hat{a}+{\hat{b}}^{†}\hat{b}+1\right)/2$, ${\hat{K}}_{+}={\hat{a}}^{†}{\hat{b}}^{†}$, ${\hat{K}}_{-}=\hat{a}\hat{b}$, [57,58],

(39) $$\hat{H}=\sum_{j=0}^{2}c_{j}{\hat{K}}_{j},$$

we immediately obtain the equation of motion for the Wigner function [37],

(40) $$i\partial_{t}W_{\rho }\left(n\right)=\left(c_{0}{\tilde{k}}_{0}-c_{1}{\tilde{k}}_{1}-c_{2}{\tilde{k}}_{2}\right)W_{\rho }\left(n\right),$$

where the first-order differential operators ${\tilde{k}}_{j}$ are defined in (16).

In the case of quadratic Hamiltonians,

(41) $$\hat{H}=\chi {\hat{K}}_{j}^{2},$$

the evolution equations take the form

(42) $$i\partial_{t}W_{\rho }\left(n\right)=\pm \chi \left(n_{j}A\left(L^{2}\right)-i{\left(n\times \tilde{k}\right)}_{j}B\left(L^{2}\right)\right){\tilde{k}}_{j}W_{\rho }\left(n\right),$$

where the sign “+” is for j=0 and the sign “−” is for j=1,2.

For instance, the equation of motion for the Hamiltonian describing Kerr-like nonlinearity [59],

(43) $$\hat{H}=\chi {\hat{K}}_{0}^{2}$$

in hyperbolic coordinates (τ,ϕ) is reduced to

(44) $$\partial_{t}W_{\rho }\left(\tau ,\phi \right)=-\chi \left(\cosh\tau A\left(L^{2}\right)+\sinh\tau \partial_{\tau }B\left(L^{2}\right)\right)\partial_{\phi }W_{\rho }\left(\tau ,\phi \right).$$

Equation (42) admit exact solutions in the following form

(45) $$W_{\rho }\left(n|t\right)=\frac{1}{2\pi }\int d\nu \left(\lambda \right)\int dn^{\prime }\Phi^{-1/2}\left(\lambda \right)P_{-1/2+i\lambda }\left(n\cdot n^{\prime }\right)Q_{\rho }\left(n^{\prime }|t\right),$$

in accordance with relations (24), where the corresponding $Q_{\rho }\left(n|t\right)\;$ functions in the basis of eigenfunctions of the ${\tilde{k}}_{j}$ operators satisfy some first-order partial differential equations. In Appendix B and Appendix B.1, we present explicit forms of $Q_{\rho }\left(n|t\right)$ for quadratic Hamiltonians possessing a discrete spectrum (43) and a continuous spectrum,

(46) $$\hat{H}=\chi {\hat{K}}_{2}^{2},$$

describing effective four-photon processes [60,61]. It is important to stress that Hamiltonians (43) and (46) are not unitary equivalent under SU(1,1) transformations and describe qualitatively different evolutions on the hyperboloid.

A comparison of the quantum and semiclassical dynamics is given in the next section.

## 5. Semiclassical Limit

The semiclassical expansion is usually performed over the inverse powers of some physical parameter (the semiclassical parameter), which acquires a large value for a given quantum system prepared in an appropriate initial state. From a mathematical perspective, the semiclassical limit for systems with the SU(1,1) symmetry corresponds to a large Bargman index, as can be observed from Equation (35). Then, ε defined in Equation (38) can be considered as a semiclassical expansion parameter whenever ε≪1. In physical realizations, this corresponds to the inverse of the difference of excitations in two-mode interaction Hamiltonians, the inverse coupling constant for the singular oscillator, etc. [28].

It is easy to see that in the semiclassical limit, the operational function (37) behaves as

(47) $$\Psi \left(L^{2}\right)≃2-\varepsilon^{2}\frac{\left(2L^{2}+1\right)}{2},$$

so that

$$A\left(L^{2}\right)=\varepsilon^{-1}+O\left(\varepsilon \right),\;B\left(L^{2}\right)=O\left(\varepsilon \right).$$

Thus, the zero-order approximation of the CR for the Wigner function (35) reads as,

(48) $$D_{L,R}^{\left(0\right)}\left({\hat{K}}_{j}\right)=\frac{1}{2}\left(\varepsilon^{-1}n_{j}\pm {\tilde{k}}_{j}\right)+O\left(\varepsilon \right),$$

while for the *Q* and *P* functions, the CRs preserve their original structure (33).

In particular, the evolution Equation (42) is reduced to the Liouville form:

(49) $$\begin{array}{ccc} \partial_{t}W_{\rho } & = & -\varepsilon^{-1}{\left\{W_{K_{j}^{2}},W_{\rho }\right\}}_{P}+O\left(\varepsilon \right), \end{array}$$

(50) $$\begin{array}{ccc} {\left\{f,g\right\}}_{P} & = & \frac{1}{\sinh\tau }\left(\partial_{\phi }f\partial_{\tau }g-\partial_{\tau }f\partial_{\phi }g\right) \end{array}$$

Here, the leading term is a first-order differential operator describing the classical dynamics, and the first-order corrections to the classical motion vanish. According to Equation (49), every point of the Wigner function evolves along the corresponding classical trajectory $n\left(t\right)=\left(\tau (t),\phi (t)\right)$,

(51) $$W_{\rho }\left(n|t\right)=W_{\rho }\left(n\left(t\right)\right),$$

leading to a deformation of the initial distribution in the course of an anharmonic dynamics. This, so-called Truncated Wigner Approximation [62,63,64,65,66,67,68,69,70,71] has been widely used in quantum systems with different symmetries for the description of short-time dynamic effects.

It is worth observing that the semiclassical parameter is inversely proportional to the representation (Bargman) index, which is consistent with the semiclassical limit of the Berezin–Toeplitz quantization approach [53,54,55]. However, its explicit form is different for every *s*-parametrized quasidistribution $W_{\rho }^{\left(s\right)}\left(n\right)$. For instance, if follows from (33) that

$$Q_{K_{0}^{2}}*Q_{\rho }={\left(D_{L}^{\left(-1\right)}\left({\hat{K}}_{0}\right)\right)}^{2}Q_{\rho }=Q_{K_{0}^{2}}Q_{\rho }+\frac{{\left(2k+1\right)}^{-1}}{\sinh\tau }\partial_{\tau }Q_{K_{0}^{2}}\partial_{\phi }Q_{\rho }+O\left(k^{-2}\right),$$

which implies that the appropriate semiclassical parameter for the *Q* function is ${\left(2k+1\right)}^{-1}$ instead of ${\left(2k-1\right)}^{-1}$ as for the Wigner function. In particular, the equations of motion for the *Q* and *P* functions expanded in powers of $\varepsilon ={\left(2k-1\right)}^{-1}$ do not acquire the form (49) in the semiclassical limit, since the first-order corrections to the Poisson brackets would be of order O(1).

**Figure 1:**
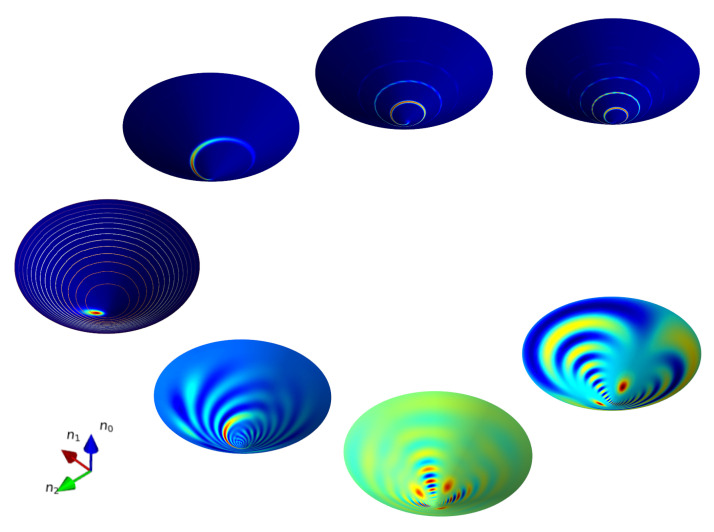
Snapshots of the Wigner function describing the evolution generated by the Hamiltonian $\hat{H}={\hat{K}}_{0}^{2}$ at times t=0,0.2,π/3, π/2 for the initial state |τ=1.5,ϕ=0〉. The upper panel and lower panels describe the semiclassical and quantum dynamics correspondingly.

In the case of evolution generated by the Hamiltonian (43), the classical equations of motion,

(52) $$\dot{\tau }=0,\;\dot{\phi }=-2k\chi \cosh\tau ,$$

describe well only the initial deformation (squeezing) of the coherent state (5) up to times $\sqrt{k}\chi t_{sem}≲1$. The early stage of squeezing of the distribution is followed by the formation of *N*-component Schrodinger cat states at χt=π/N, along with a typical interference pattern, the description of which is beyond the semiclassical approximation. In Figure 1 we plot the semiclassical (51) and quantum (45), (A14) evolution of the Wigner function of an initial coherent state (5) under the action of the Hamiltonian (43).

**Figure 2:**
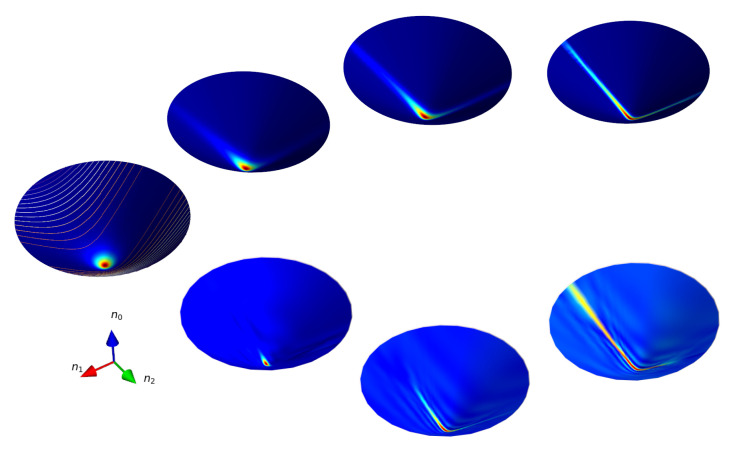
Snapshots of the Wigner function describing the evolution generated by the Hamiltonian $\hat{H}={\hat{K}}_{2}^{2}$ at times t=0,0.2,1,2 for the initial state |τ=0,ϕ=0〉. The upper panel and lower panels describe the semiclassical and quantum dynamics correspondingly.

The evolution generated by the Hamiltonian (46) is very different from that induced by (43). The classical trajectories are obtained from

(53) $$\begin{array}{ccc} \dot{\phi } & = & 2k\chi \sin^{2}\phi \cosh\tau , \end{array}$$

(54) $$\begin{array}{ccc} \dot{\tau } & = & -2k\chi \sinh\tau \sin\phi \cos\phi , \end{array}$$

preserving the integral of motion $E=k^{2}{\left(\sinh\tau \sin\phi \right)}^{2}$. The initial coherent state |τ=0,ϕ=0〉 located at the origin of the hyperboloid suffers a deformation in the vicinity of the minimum of the classical potential (mainly in the valley along the axis $n_{2}$),

(55) $$\langle n|{\hat{K}}_{2}^{2}|n\rangle \approx k^{2}\sinh^{2}\tau \sin^{2}\phi ,$$

according to Equations (53) and (54) for $\chi t_{sem}≲1$ at long time scales. In other words, the quantum evolution of the initial distribution corresponding to the coherent state located at the minimum of the potential (55) is well simulated by semiclassical dynamics. In Figure 2, we plot the semiclassical (51) and quantum (45), (A29) evolution of the Wigner function of an initial coherent state (5) located at τ=0 under the action of the Hamiltonian (46).The main difference between the semiclassical and the quantum evolutions of the Wigner function is the appearance of small amplitude ripplings and a slight bending toward the axis $n_{1}$ in the latter. Observe that in this case, there is no emergence of the Schrodinger cat states. It is worth noting that the long-time quantum evolution of distributions that are not located initially at the origin of the hyperboloid may significantly differ from its classical counterpart.

## 6. Conclusions

We have obtained the correspondence rules for the *s*-parametrized distributions in the hyperbolic phase space. The relations (33) and (35) allow us to deduce the exact evolution equations for polynomial Hamiltonians on the SU(1,1) algebra generators. Those equations can be solved in a systematic way for diagonal quadratic Hamiltonians (41).

The semiclassical limit corresponds to the large values of the Bargman index, which labels the discrete irreducible representations of the SU(1,1) group. The leading order term of the semiclassical expansion of the evolution equation for the Wigner function is reduced to the Poisson brackets on the hyperboloid. Surprisingly, the exact long-term non-harmonic evolution of certain states generated by the continuous-spectrum Hamiltonian (46) is well described in the semiclassical approximation (49). This contradicts our intuition of a typical behavior of phase-space distributions, the evolution of which is governed by non-linear (on the group generators) Hamiltonians, as occurs in case of the discrete-spectrum Hamiltonian (43), where the emergence of the Schrodinger cat states cannot be explained from the classical point of view.

## Data Availability

Not applicable.

## Appendix A

In this Appendix, we apply the similarity transformation generated by the differential operator (23) to the components of the pseudo-Bloch vector (6), i.e., we compute $\Phi^{1/2}\left(L^{2}\right)n_{j}\Phi^{-1/2}\left(L^{2}\right)$, j=0,1,2.

We outline the procedure on the example of $n_{0}=\cosh\tau$. Applying

$\Phi^{1/2}\left(L^{2}\right)\cosh\tau \Phi^{-1/2}\left(L^{2}\right)$ to the harmonic function (11), and making use of the recurrence relation for the associated Legendre polynomials

(A1) $$\cosh\tau P_{\nu }^{n}\left(\cosh\tau \right)=\frac{\nu -n+1}{2\nu +1}P_{\nu +1}^{n}\left(\cosh\tau \right)+\frac{\nu +n}{2\nu +1}P_{\nu -1}^{n}\left(\cosh\tau \right),$$

we get

$$\begin{array}{ccc} & & \Phi^{1/2}\left(L^{2}\right)\cosh\tau \Phi^{-1/2}\left(L^{2}\right)u_{n}^{\lambda }\left(n\right)=\frac{\Phi^{-1/2}\left(\lambda \right)}{2i\lambda }\frac{{\left(-1\right)}^{n}\Gamma \left(\frac{1}{2}+i\lambda \right)}{\Gamma \left(\frac{1}{2}+i\lambda +n\right)}e^{in\phi }\times \\ & & \times \left[\left(\frac{1}{2}+i\lambda -n\right)\Phi \left(\lambda -i\right)P_{\frac{1}{2}+i\lambda }^{n}\left(\cosh\tau \right)-\left(\frac{1}{2}-i\lambda -n\right)\Phi \left(\lambda +i\right)P_{-\frac{3}{2}+i\lambda }^{n}\left(\cosh\tau \right)\right], \end{array}$$

where Φ(λ) is defined in (27). Now, expressing the associated Legendre functions that appear in the above expression in terms of the conic function $P_{\frac{1}{2}+i\lambda }^{n}\left(\cosh\tau \right)$, by means of the relations

(A2) $$\begin{array}{ccc} P_{\frac{1}{2}+i\lambda }^{n}\left(\cosh\tau \right) & = & \frac{1}{\frac{1}{2}+i\lambda -n}\left[\left(\frac{1}{2}+i\lambda \right)\cosh\tau +\sinh\tau \partial_{\tau }\right]P_{-\frac{1}{2}+i\lambda }^{n}\left(\cosh\tau \right), \end{array}$$

(A3) $$\begin{array}{ccc} P_{-\frac{3}{2}+i\lambda }^{n}\left(\cosh\tau \right) & = & \frac{1}{-\frac{1}{2}+i\lambda +n}\left[\left(-\frac{1}{2}+i\lambda \right)\cosh\tau -\sinh\tau \partial_{\tau }\right]P_{-\frac{1}{2}+i\lambda }^{n}\left(\cosh\tau \right), \end{array}$$

we arrive at the following transformation rule:

(A4) $$\Phi^{1/2}\left(L^{2}\right)\cosh\tau \Phi^{-1/2}\left(L^{2}\right)u_{n}^{\lambda }\left(n\right)=\left[a\left(\lambda \right)\cosh\tau +b\left(\lambda \right)\sinh\tau \partial_{\tau }\right]u_{n}^{\lambda }\left(n\right)$$

where

(A5) $$\begin{array}{ccc} a(\lambda ) & = & \frac{\Phi^{-1/2}\left(\lambda \right)}{2i\lambda }\left[\left(\frac{1}{2}+i\lambda \right)\Phi^{1/2}\left(\lambda -i\right)+\left(-\frac{1}{2}+i\lambda \right)\Phi^{1/2}\left(\lambda +i\right)\right], \end{array}$$

(A6) $$\begin{array}{ccc} b(\lambda ) & = & \frac{\Phi^{-1/2}\left(\lambda \right)}{2i\lambda }\left[\Phi^{1/2}\left(\lambda -i\right)-\Phi^{1/2}\left(\lambda +i\right)\right]. \end{array}$$

The product $\Phi^{-1/2}\left(\lambda \right)\Phi^{1/2}\left(\lambda \pm i\right)$ can be conveniently rewritten as

$$\begin{array}{ccc} \Phi^{-1/2}\left(\lambda \right)\Phi^{1/2}\left(\lambda -i\right) & = & \frac{\varphi_{\frac{1}{2}+i\lambda }}{1-\varepsilon (1/2+i\lambda )},\\ \Phi^{-1/2}\left(\lambda \right)\Phi^{1/2}\left(\lambda +i\right) & = & \frac{\varphi_{-\frac{1}{2}+i\lambda }}{1+\varepsilon (-1/2+i\lambda )},\\ \varphi_{\frac{1}{2}+i\lambda } & = & \sqrt{1-\varepsilon^{2}\left(1/2+i\lambda \right)}, \end{array}$$

where $\varepsilon ={\left(2k-1\right)}^{-1}$. Observing that $\sinh\tau \partial_{\tau }=i{\left(n⋊\tilde{k}\right)}_{0}$, we represent the required transformation in the vector form

(A7) $$\Phi^{1/2}\left(L^{2}\right)n_{0}\Phi^{-1/2}\left(L^{2}\right)=a\left(\lambda \right)n_{0}+ib\left(\lambda \right){\left(n⋊\tilde{k}\right)}_{0},$$

where

(A8) $$a\left(\nu \right)=\frac{1}{2\nu +1}\left[\frac{\left(\nu +1\right)\varphi_{\nu +1}}{1-\varepsilon (\nu +1)}+\frac{\nu \varphi_{\nu }}{1+\varepsilon \nu }\right],\;\;\;\;\;\;b\left(\nu \right)=\frac{1}{2\nu +1}\left[\frac{\varphi_{\nu +1}}{1-\varepsilon (\nu +1)}+\frac{\varphi_{\nu }}{1+\varepsilon \nu }\right]$$

In a very similar way, one obtains

(A9) $$\Phi^{1/2}\left(L^{2}\right)n_{j}\Phi^{-1/2}\left(L^{2}\right)=a\left(\lambda \right)n_{j}+ib\left(\lambda \right){\left(n⋊\tilde{k}\right)}_{j}.$$

The transformation of $i{\left(n⋊\tilde{k}\right)}_{j}$ can be simplified by making use of the orthogonality relation (18), obtaining

(A10) $$\Phi^{1/2}\left(L^{2}\right)i{\left(n⋊\tilde{k}\right)}_{j}\Phi^{-1/2}\left(L^{2}\right)=i\left[a(\lambda )-b(\lambda )\right]{\left(n⋊\tilde{k}\right)}_{j}-\left(\lambda^{2}+\frac{1}{4}\right)b\left(\lambda \right)n_{j}.$$

Combining (A9) and (A10) in the correspondence rule for the *P*-function (33),

(A11) $$\begin{array}{c} \Phi^{1/2}\left(L^{2}\right)\left[\left(k-1\right)n_{j}-\frac{i}{2}{\left(n⋊\tilde{k}\right)}_{j}\right]\Phi^{-1/2}\left(L^{2}\right)u_{n}^{\lambda }\left(n\right)=\\ \frac{1}{2}\left\{\left[2\left(k-1\right)a\left(\lambda \right)+\left(\lambda^{2}+\frac{1}{4}\right)b\left(\lambda \right)\right]n_{j}+i\left[\left(2k-1\right)b\left(\lambda \right)-a\left(\lambda \right)\right]{\left(n⋊\tilde{k}\right)}_{j}\right\}u_{n}^{\lambda }\left(n\right)=\\ \frac{1}{2}\left\{n_{j}\left[\frac{1}{2\varepsilon }\Psi \left(L^{2}\right)-\frac{\varepsilon }{2}\Psi^{-1}\left(L^{2}\right)\right]-i\varepsilon {\left(n⋊\tilde{k}\right)}_{j}\Psi^{-1}\left(L^{2}\right)\right\}u_{n}^{\lambda }\left(n\right) \end{array}$$

and introducing (36) and (37), we finally arrive at expression (35).

## Appendix B

Taking into account the expressions for the elements of the D-algebra (30)–(33), we immediately obtain the evolution equation for the $Q_{\rho }\left(n\right)$ function generated by the Hamiltonian (43):

(A12) $$\partial_{t}Q_{\rho }\left(n\right)=-\chi \left(2k\cosh\tau +\sinh\tau \partial_{\tau }\right)\partial_{\phi }Q_{\rho }\left(n\right).$$

The explicit expression for Q(n|t) in case of an initial coherent state (5) $|n_{0}\rangle$ can be easily obtained by a direct computation as follows:

(A13) $$\begin{array}{ccc} Q_{\rho }\left(n|t\right) & = & |\langle n_{0}|e^{-i\chi {\hat{K}}_{0}^{2}}|n_{0}{\rangle |}^{2} \end{array}$$

(A14) $$\begin{array}{ccc} & = & \cosh^{-2k}\frac{\tau }{2}\cosh^{-2k}\frac{\tau_{0}}{2}|\sum_{m}\gamma_{m}{\left(\tanh\frac{\tau }{2}\tanh\frac{\tau_{0}}{2}\right)}^{m}e^{i\left(\phi -\phi_{0}\right)m-it{\left(m+k\right)}^{2}}{|}^{2},\\ \gamma_{m} & = & \frac{\Gamma (m+2k)}{m!\Gamma (2k)}, \end{array}$$

since ${\hat{K}}_{0}$ is diagonal in the basis (3). However, it is instructive to solve Equation (A12) in a systematic way. The expansion coefficients of Q(n|t) in Fourier series (eigenfunctions of the ${\tilde{k}}_{0}$ operator)

(A15) $$Q_{\rho }\left(n|t\right)=\sum_{n=-\infty }^{\infty }c_{n}\left(\tau |t\right)e^{in\phi },$$

satisfy the following first-order differential equation:

(A16) $$\partial_{t}c_{n}+in\;\sinh\tau \partial_{\tau }c_{n}=-in\;2k\cosh\tau c_{n},$$

where the initial condition according to (9) is

(A17) $$\begin{array}{ccc} c_{n}\left(\tau |0\right) & = & \frac{1}{2\pi }\int_{0}^{2\pi }d\phi e^{-in\phi }{\left(\frac{1+n\cdot n_{0}}{2}\right)}^{-2k} \end{array}$$

(A18) $$\begin{array}{ccc} & = & \cosh^{-2k}\frac{\tau }{2}\cosh^{-2k}\frac{\tau_{0}}{2}e^{-i\phi_{0}n}\sum_{m}\gamma_{m}\gamma_{m-n}{\left(\tanh\frac{\tau }{2}\tanh\frac{\tau_{0}}{2}\right)}^{2m-n}, \end{array}$$

which can be also represented as

$$c_{n}\left(\tau |0\right)=2^{2k}{\left(-1\right)}^{n}e^{-i\phi_{0}n}\frac{\Gamma (2k)}{\Gamma (2k-n)}{\left(\cosh\tau +\cosh\tau_{0}\right)}^{-2k}P_{-2k}^{n}\left(\frac{1+\cosh\tau \cosh\tau_{0}}{\cosh\tau +\cosh\tau_{0}}\right),$$

where $P_{-2k}^{n}\left(x\right)$ is the Legendre function of the first kind [56].

Then, the solution of (A16) has the form

(A19) $$\begin{array}{ccc} c_{n}\left(\tau |t\right) & = & \frac{1}{\sinh^{2k}\tau }\sinh^{2k}\left(\tau_{n}\left(t\right)\right)c_{n}\left(0,\tau_{n}\left(t\right)\right), \end{array}$$

(A20) $$\begin{array}{ccc} \tau_{n}\left(t\right) & = & 2\mathrm{arctanh}(e^{-int}\tanh\tau /2). \end{array}$$

Substituting (A19) into (A15), we return after simple algebra to the expression (A14).

### Appendix B.1

The evolution equation for the $Q_{\rho }\left(n\right)$ function generated by the Hamiltonian (46) is

(A21) $$i\partial_{t}Q_{\rho }\left(n\right)=-\left(2kn_{2}+i{\left(n\times \tilde{k}\right)}_{2}\right){\tilde{k}}_{2}Q_{\rho }\left(n\right).$$

In the canonical variables X=(x,y)

$$\begin{array}{ccc} y & = & \ln(\cosh\tau +\sinh\tau \cos\phi ),\;x=\sinh\tau \sin\phi ,\\ {\left\{y,x\right\}}_{P} & = & 1, \end{array}$$

with ${\left\{.,.\right\}}_{P}$ being the Poisson brackets defined in (50), and Equation (A21) acquires the form

(A22) $$\partial_{t}Q_{\rho }\left(X\right)=\left(2kx+\left(x^{2}+1\right)\partial_{x}+x\partial_{y}\right)\partial_{y}Q_{\rho }\left(X\right).$$

The expansion coefficients in the Fourier integral

(A23) $$Q_{\rho }\left(X|t\right)=\int d\alpha e^{i\alpha y}c_{\alpha }\left(x|t\right)$$

satisfy the equation

(A24) $$\partial_{t}c_{\alpha }+i\alpha \left(x^{2}+1\right)\partial_{x}c_{\alpha }=-i\alpha x\left(-2k+i\alpha \right)c_{\alpha },$$

where $c_{\alpha }\left(x|0\right)$ corresponds to the initial coherent state |τ=0,ϕ=0〉, with

$$Q_{\rho }\left(\tau ,\phi |0\right)={\left(\frac{1+\cosh\tau }{2}\right)}^{-2k},$$

are

(A25) $$\begin{array}{ccc} c_{\alpha }\left(x|0\right) & = & \frac{2^{2k}}{2\pi }\int dye^{-i\alpha y}{\left(\frac{1}{2}e^{-y}\left(1+e^{2y}+x^{2}\right)+1\right)}^{-2k} \end{array}$$

(A26) $$\begin{array}{ccc} & = & 2^{2k+1}e^{-i\alpha A+\pi \alpha }\frac{{\left(-1\right)}^{k}}{{\left(4x^{2}\right)}^{k}}\frac{\Gamma (2k-i\alpha )}{\Gamma (2\alpha )}Q_{2k-1}^{i\alpha }\left(-i/x\right), \end{array}$$

where $Q_{2k-1}^{i\alpha }\left(-i/x\right)$ is the Legendre function of the second kind [56] and $\tanh A=x^{2}{\left(x^{2}+2\right)}^{-1}$.

The solution of Equation (A24) takes the form

(A27) $$\begin{array}{ccc} c_{\alpha }\left(x|t\right) & = & {\left(1+x^{2}\right)}^{k+i\alpha /2}{\left(1+\tan^{2}\chi_{\alpha }\left(t\right)\right)}^{-k-i\alpha /2}c_{\alpha }\left(\tan\chi_{\alpha }\left(t\right)|0\right), \end{array}$$

(A28) $$\begin{array}{ccc} \chi_{\alpha }\left(t\right) & = & \arctan(x)+i\alpha t, \end{array}$$

leading finally to the following expression for the evolved *Q* function in variables (x,y):

(A29) $$Q_{\rho }\left(X|t\right)=\int d\alpha e^{i\alpha y}{\left(1+x^{2}\right)}^{k+i\alpha /2}{\left(1+\tan^{2}\chi_{\alpha }\left(t\right)\right)}^{-k-i\alpha /2}c_{\alpha }\left(\tan\chi_{\alpha }\left(t\right)|0\right).$$

## References

[1] Zachos C.K., Fairle D.B., Curtright T.L. (2005). Quantum Mechanics in Phase Space.

[2] Osorio de Almeida A.M. (1998). The Weyl representation in classical and quantum mechanics. Phys. Rep..

[3] Schroeck F. (1996). Quantum Mechanics on Phase Space.

[4] Moyal J.E. (1949). Quantum mechanics as a statistical theory. Proc. Camb. Phil. Soc..

[5] Bayen F., Flato M., Fronsdal C., Lichnerowicz A., Sternheimer D. (1978). Deformation theory and quantization. II. Physical applications. Ann. Phys. N. Y..

[6] Stratonovich R.L. (1956). On distributions in representation space. Sov. Phys. JETP.

[7] Brif C., Mann A. (1999). Phase-space formulation of quantum mechanics and quantum-state reconstruction for physical systems with Lie-group symmetries. Phys. Rev. A.

[8] Chaturvedi S., Ercolessi E., Marmo G., Morandi G., Mucunda N., Simon R. (2006). Wigner–Weyl correspondence in quantum mechanics for continuous and discrete systems—A Dirac-inspired view. J. Phys. A Math. Gen..

[9] Mucunda N., Marmo G., Zampini A., Chaturvedi S., Simon R. (2005). Wigner–Weyl isomorphism for quantum mechanics on Lie groups. J. Math. Phys..

[10] Tilma T., Everitt M.J., Samson J.H., Munro W.J., Nemoto K. (2016). Wigner Functions for Arbitrary Quantum Systems. Phys. Rev. Lett..

[11] Onofri E. (1975). A note on coherent state representations of Lie groups. J. Math. Phys..

[12] Belchev B., Walton M.A. (2009). On Wigner functions and a damped star product in dissipative phase-space quantum mechanics. Ann. Phys..

[13] Arecchi F.T., Courtens E., Gilmore R., Thomas H. (1972). Atomic Coherent States in Quantum Optics. Phys. Rev. A.

[14] Gilmore R., Bowden C.M., Narducci L.M. (1975). Classical-quantum correspondence for multilevel systems. Phys. Rev. A.

[15] Zueco D., Calvo I. (2007). Bopp operators and phase-space spin dynamics: Application to rotational quantum Brownian motion. J. Phys. A.

[16] Klimov A.B. (2002). Exact evolution equations for SU(2) quasidistribution functions. J. Math. Phys..

[17] Klimov A.B., Espinoza P. (2002). Moyal-like form of the star product for generalized SU(2) Stratonovich-Weyl symbols. J. Phys. A.

[18] Rios P.M., Straume E. (2014). Symbol Correspondences for Spin Systems.

[19] Koczor B., Zeier R., Glaser S.J. (2019). Self-trapped quantum balls in binary Bose-Einstein condensates. J. Phys. A.

[20] Amiet J.-P., Cibilis M.B. (1991). Description of quantum spin using functions on the sphere *S*^2^. J. Phys. A Math. Gen.

[21] Klimov A.B., Romero J.L. (2008). A generalized Wigner function for quantum systems with the SU(2) dynamical symmetry group. J. Phys. A.

[22] Plebanski J.F., Przanowski M., Tosiek J., Turrubiates F. (2000). Remarks on Deformation Quantization on the Cylinder. J. Acta Phys. Pol. B.

[23] Rigas I., Sanchez-Soto L.L., Klimov A.B., Rehacek J., Hradil Z. (2011). Orbital angular momentum in phase space. Ann. Phys..

[24] Martins A.C.N., Klimov A.B., de Guise H. (2019). Correspondence rules for Wigner functions over SU(3)/U(2). J. Phys. A.

[25] Bopp F. (1956). Is quantum mechanics a particular classical statistical mechanics?. Ann. Inst. H. Poincare.

[26] Zhang W.-M., Feng D.H., Gilmore R. (1990). Coherent states: Theory and some applications. Rev. Mod. Phys..

[27] Klimov A.B., Chumakov S.M. (2009). A Group-TheoreticalApproach to Quantum Optics.

[28] Perelomov A. (1986). Generalized Coherent States and Their Applications.

[29] Gazeau J.P. (2009). Coherent States in Quantum Physics.

[30] Wigner E.P. (1932). On the Quantum Correction For Thermodynamic Equilibrium. Phys. Rev..

[31] Hillery M., O’Connell R.F., Scully M.O., Wigner E.P. (1984). Distribution functions in physics: Fundamentals. Phys. Rep..

[32] Lee H.W. (1995). Theory and application of the quantum phase-space distribution functions. Phys. Rep..

[33] Gadella M. (1995). Moyal Formulation of Quantum Mechanics. Fortschr. Phys..

[34] Agarwal G.S. (1981). Relation between atomic coherent-state representation, state multipoles, and generalized phase-space distributions. Phys. Rev. A.

[35] Dowling J.P., Agarwal G.S., Schleich W.P. (1994). Wigner distribution of a general angular-momentum state: Applications to a collection of two-level atoms. Phys. Rev. A.

[36] Várilly J.C., Gardia-Bondía J.M. (1989). The moyal representation for spin. Ann. Phys..

[37] Gerry C.C. (1985). Dynamics of SU(1,1) coherent states. Phys. Rev. A.

[38] Gerry C.C. (1991). Correlated two-mode SU(1, 1) coherent states: Nonclassical properties. J. Opt. Soc. Am. B.

[39] Yurke B., McCall S.L., Klauder J.R. (1986). SU(2) and SU(1,1) interferometers. Phys. Rev. A.

[40] Jing J., Liu C., Zhou Z., Ou Z.Y., Zhang W. (2011). Realization of a nonlinear interferometer with parametric amplifiers. Appl. Phys. Lett..

[41] Hudelist F., Kong J., Liu C., Jing J., Ou Z.Y., Zhang W. (2014). Quantum metrology with parametric amplifier-based photon correlation interferometers. Nat. Commun..

[42] Orłowski A., Wódkiewicz K. (1990). On the SU(1, 1) Phase-space Description of Reduced and Squeezed Quantum Fluctuations. J. Mod. Opt..

[43] Brif C. (1997). SU (2) and SU (1, 1) algebra eigenstates: A unified analytic approach to coherent and intelligent states. Int. J. Theor. Phys..

[44] Wodkiewicz K., Eberly J.H. (1985). Coherent states, squeezed fluctuations, and the SU(2) am SU(1,1) groups in quantum-optics applications. J. Opt. Soc. Am. B.

[45] Klimov A.B., Seyfarth U., de Guise H., Sánchez-Soto L.L. (2021). SU(1, 1) covariant s-parametrized maps. J. Phys. A.

[46] del Olmo M.A., Gazeau J.P. (2020). Covariant integral quantization of the unit disk. J. Math. Phys..

[47] Akhtar N., Sanders B.C., Xianlongl G. (2022). Sub-Planck phase-space structure and sensitivity for SU(1,1) compass states. Phys. Rev. A.

[48] Klimov A.B., Romero J.L., de Guise H. (2017). Generalized SU(2) covariant Wigner functions and some of their applications. J. Phys. A Math. Theor..

[49] Glauber R.J. (1963). Coherent and Incoherent States of the Radiation Field. Phys. Rev..

[50] Sudarshan E.C.G. (1963). Equivalence of Semiclassical and Quantum Mechanical Descriptions of Statistical Light Beams. Phys. Rev. Lett..

[51] Husimi K. (1940). Some Formal Properties of the Density Matrix. Proc. Phys. Math. Soc. Jpn..

[52] Kano Y. (1965). A New Phase-Space Distribution Function in the Statistical Theory of the Electromagnetic Field. J. Math. Phys..

[53] Berezin F.A. (1975). General concept of quantization. Commun. Math. Phys..

[54] Berezin F.A. (1974). Quantization. Quantization. Math. USSR-Izv..

[55] Schlichenmaier M. (2010). Berezin–Toeplitz quantization for compact Kähler manifolds. A review of results. Adv. Math. Phys..

[56] Erdélyi A., Magnus W., Oberhettinger F., Tricomi F.G. (1955). Higher Transcendental Functions.

[57] Hillery M., Zubairy M.S. (1982). Path-integral approach to problems in quantum optics. Phys. Rev. A.

[58] Gerry C.C., Welch R.E. (1992). Dynamics of a two-mode two-photon Jaynes–Cummings model interacting with correlated SU(1, 1) coherent states. J. Opt. Soc. Am. B.

[59] Banerji J., Agarwal G.S. (1999). Revival and fractional revival in the quantum dynamics of SU(1,1) coherent states. Phys. Rev. A.

[60] Tombesi P., Mecozzi A. (1988). Four-photon squeezed states: An exactly solvable model. Phys. Rev. A.

[61] Gerry C.C., Kiefer J. (1990). Classical dynamics and ground-state phase transitions of a model SU(1,1) Hamiltonian. Phys. Rev. A.

[62] Ballentine L.E., Yang Y., Zibin J.P. (1994). Inadequacy of Ehrenfest’s theorem to characterize the classical regime. Phys. Rev. A.

[63] Heller E.J. (1976). Wigner phase space method: Analysis for semiclassical applications. Chem. Phys..

[64] Heller E.J. (1977). Phase space interpretation of semiclassical theory. Chem. Phys..

[65] Heller E.J., Reimers J.R., Drolshagen G. (1987). Classical and semiclassical approximations for incoherent neutron scattering. Phys. Rev. A..

[66] Davis M.J., Heller E.J. (1984). Comparisons of classical and quantum dynamics for initially localized states. J. Chem. Phys..

[67] Kinsler P., Drummond P.D. (1993). Limits to squeezing and phase information in the parametric amplifier. Phys. Rev. A.

[68] Drobny G., Jex I. (1992). Quantum properties of field modes in trilinear optical processes. Phys. Rev. A..

[69] Drobny G., Bandilla A., Jex I. (1996). Nondegenerate parametric interactions and nonclassical effects. Phys. Rev. A.

[70] Klimov A.B., Espinoza P. (2005). Classical evolution of quantum fluctuations in spin-like systems: Squeezing and entanglement. J. Opt. B Quant. Semiclass. Opt..

[71] Polkovnikov A. (2010). Phase space representation of quantum dynamics. Ann. Phys..

